# Differing Susceptibilities to Certain Microbicidal Chemistries among Three Representative Enveloped Viruses

**DOI:** 10.3390/microorganisms12030535

**Published:** 2024-03-07

**Authors:** Tanya Kapes, Charles Quinn, Andrew Eli Cragun, Taylor House, Raymond W. Nims, S. Steve Zhou

**Affiliations:** 1 Microbac Laboratories, Inc., Sterling, VA 20164, USA; tanya.kapes@microbac.com (T.K.); charles.quinn@microbac.com (C.Q.);; 2 Syner-G Biopharma, Boulder, CO 80503, USA

**Keywords:** bovine viral diarrhea virus, ethanol, liquid inactivation, low pH inactivation, peracetic acid, quaternary ammonium compound, SARS-CoV-2, sodium hypochlorite, vaccinia virus

## Abstract

Three lipid-enveloped viruses (bovine viral diarrhea virus [BVDV], vaccinia virus, and severe acute respiratory syndrome coronavirus 2 [SARS-CoV-2]) were evaluated in side-by-side liquid inactivation efficacy studies of low pH (3.0 to 3.1) treatment and of the non-formulated microbicidal actives sodium hypochlorite (100 ppm), ethanol (70%), quaternary ammonium compound BTC^®^ 835 (100 ppm), and peracetic acid (100 ppm). Low pH was evaluated at 10 and 60 min contact times, and the microbicides were evaluated at 1 min contact time at room temperature per the ASTM E1052 standard. In each case, 5% animal serum was included in the viral inoculum as a challenge soil load. The three viruses displayed similar susceptibility to sodium hypochlorite and ethanol, with complete inactivation resulting. Significant differences in susceptibility to BTC^®^ 835 and peracetic acid were identified, with the ordering of the three viruses for susceptibility to BTC^®^ 835 being SARS-CoV-2 > vaccinia virus = BVDV, and the ordering for peracetic acid being vaccinia virus > SARS-CoV-2 > BVDV. The ordering for susceptibility to low pH treatment (60 min contact time) was vaccinia virus > SARS-CoV-2 > BVDV. Not all enveloped viruses display equivalent susceptibilities to inactivation approaches. For the chemistries evaluated here, BVDV appears to represent a worst-case enveloped virus.

## 1. Introduction

Viruses are causative agents for many human and animal infectious diseases, and some are implicated in other illnesses such as cancer. In the biopharmaceutical industry, viral contamination control is also a critical aspect for product quality and safety. Public hygienic measures such as antisepsis, disinfection, and sanitization are important in preventing the spread of viral pathogens; and they are especially valuable in cases where specific pharmaceutical interventions may be absent or limited. 

When the efficacy of antiseptics, disinfectants, and other infection control products is evaluated, laboratory testing utilizing infectious model viruses, as available, is commonly used. Additionally, efficacy may be extrapolated from one virus to other virus(es), either based on the literature or an observed general trend of the “hierarchy” of the susceptibility of various microorganisms. This paradigm has been widely used by the infection prevention and control (IPAC) community and has been immensely valuable during novel virus outbreaks, such as the recent SARS-CoV-2 pandemic. The paradigm was based on prior classifications by Spaulding [1] and Klein and Deforest [2], before evolving eventually into the hierarchy of the susceptibility of pathogens to microbicidal actives [3,4,5,6]. The value of the paradigm is in its ability to best predict the efficacy of differing microbicidal chemistries for inactivating emerging/re-emerging viral pathogens, for which empirical efficacy data may not yet be available [5]. 

**Figure 1:**
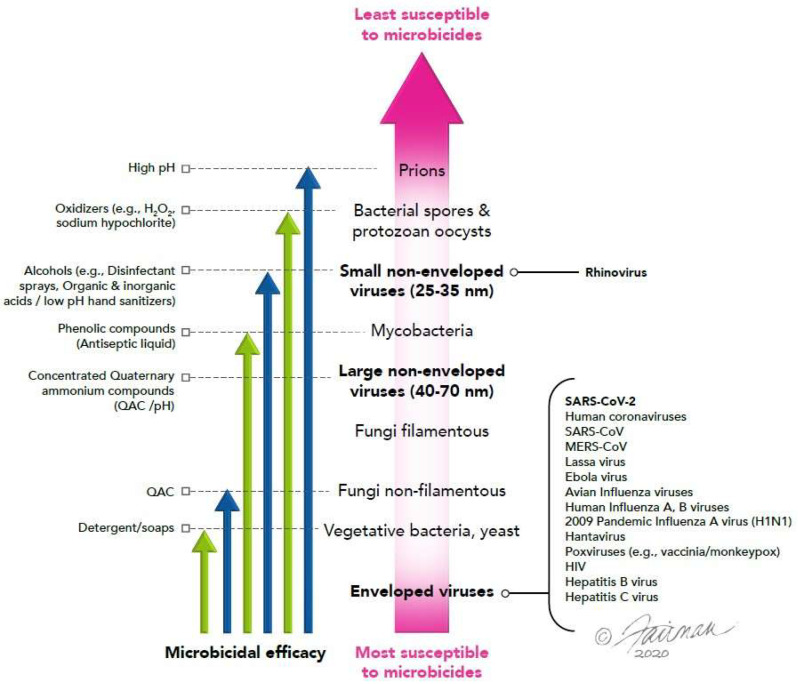
A version of the hierarchy of susceptibility of pathogens to microbicides. Figure from [6].

Most versions of the hierarchy paradigm (Figure 1) also contain information on which microbicidal chemistries should inactivate the different pathogen types. While quite useful, it may not be realized by all that there are exceptions to the hierarchy paradigm. In particular, there is danger in assuming that the various microbicidal chemistries will inactivate all pathogens within a given pathogen level to an equal degree. For example, one might expect that all small non-enveloped viruses will display similar susceptibilities to alcohols, oxidizing agents, high pH, or aldehydes. In reality, there have been identified substantial differences in susceptibility even among this one pathogen group, as demonstrated by Zhou [7], and even within a given viral family within this category of pathogens [8].

Despite a wealth of information on the similarities or differences in the susceptibility of non-enveloped viruses, there has been limited research on side-by-side comparisons of enveloped viruses to chemical inactivation. Enveloped viruses universally have been considered the most susceptible pathogens to microbicidal actives among versions of the hierarchy paradigm [1,2,3,4,5,6]; it is commonly assumed that enveloped viruses will react similarly to various microbicidal actives. The reliability of this assumption, however, has not been rigorously tested. Additionally, if significant differences in the susceptibility among enveloped viruses to certain microbicidal actives do exist, are there specific enveloped virus(es) that may be considered a worst-case virus?

In this article, we have attempted to answer these questions by running side-by-side liquid (suspension) efficacy studies utilizing five commonly used types of microbicidal actives (acid, sodium hypochlorite, ethanol, quaternary ammonium compound, and peracetic acid) against three enveloped viruses: severe acute respiratory syndrome coronavirus 2 (SARS-CoV-2, family *Coronaviridae*), vaccinia virus (family *Poxviridae*), and bovine viral diarrhea virus (BVDV, family *Flaviviridae*).

## 2. Materials and Methods

### 2.1. Challenge Viruses and Host Cell Lines

Bovine viral diarrhea virus, strain NADL, was obtained from American BioResearch Laboratories (Seymour, TN, USA) and vaccinia virus, strain MVA, ATCC VR-1508, was sourced from American Type Culture Collection (Manassas, VA, USA). Severe acute respiratory syndrome coronavirus 2 (SARS-CoV-2), isolate USA-WA1/2020, NR-52281, was obtained from the BEI Resources (Manassas, VA, USA). The host cell lines used were Madin–Darby bovine kidney (MDBK, ATCC CCL-22) for BVDV, Syrian hamster kidney (BHK-21, ATCC CCL-10) for vaccina virus, and African green monkey kidney (Vero E6, ATCC CRL-1586) for SARS-CoV-2, in each case obtained from American Type Culture Collection. Cell culture media used for the MDBK, BHK-21, and Vero E6 cell lines were Minimum Essential Medium (MEM) + 10% horse serum, RPMI 1640 + 10% newborn calf serum, and MEM + 10% fetal bovine serum (FBS), respectively.

### 2.2. Microbicides and Neutralizers Used

The microbicidal chemicals evaluated in this study included sodium hypochlorite (Clorox, Oakland, CA, USA), ethanol (ThermoFisher, Fair Lawn, NJ, USA), peracetic acid (Sigma-Aldrich, Saint Louis, MO, USA), hydrochloric acid (Fisher Scientific, Fair Lawn, NJ, USA), and a quaternary ammonium compound, BTC^®^ 835 (alkyl dimethyl benzyl ammonium chloride) (Stepan, Northfield, IL, USA). In each case, sterile deionized water was used to prepare dilutions to use concentrations (100 ppm for sodium hypochlorite, peracetic acid, and BTC^®^ 835; 70% for ethanol; and 0.5 N for hydrochloric acid). The chemical neutralizers used to quench the virucidal activities of the actives to enable time kinetics of inactivation to be determined included Minimum Essential Medium (MEM) + 10% FBS + 0.5% Na_2_S_2_O_3_ (for sodium hypochlorite), MEM + 10% FBS (for ethanol), MEM + 10% FBS + 1% NaHCO_3_ +1% HEPES + 0.5% Na_2_S_2_O_3_ + 0.01 N NaOH (for peracetic acid and low pH), and MEM + 10% FBS + 0.5% Polysorbate 80 + 0.5% lecithin (for BTC^®^ 835). For use with BVDV, the neutralizers were prepared using horse serum instead of FBS.

### 2.3. Suspension Inactivation Testing Methodology

The liquid (suspension) viral inactivation studies were performed per ASTM E1052-20 [9]. The virucidal efficacies of 100 ppm sodium hypochlorite, 70% ethanol, 100 ppm BTC^®^ 835, and 100 ppm peracetic acid were determined as follows. For each replicate run, 0.3 mL of challenge virus (containing 5% horse serum for BVDV and 5% FBS for vaccinia virus and SARS-CoV-2 as organic load) was spiked into 2.7 mL of the test microbicide and held for 1 min at room temperature (20–22 °C). After the contact time, the reaction mixture was neutralized 1:1 *v*/*v* with the appropriate chemical neutralizer (described above). The post-neutralized solutions were then 10-fold serially diluted with MEM + 2% horse serum for BVDV and MEM + 2% FBS for vaccinia virus and SARS-CoV-2, and inoculated onto the appropriate host cells for each virus for determining the infectious titers using the Tissue Culture Infectious Dose 50% (TCID_50_) assay. The inoculated host cells were incubated at 36 °C with 5% CO_2_ for 5–11 days and scored for cytopathic effects. The infectious virus titers were calculated using the Spearman–Kärber formula [10].

A virus recovery control was performed by combining the virus and a mock solution (deionized water for low pH treatment or the dilution media described above for all other chemistries), held for the contact time, and neutralized using the same neutralizer as used for the microbicidal chemistry test runs. Therefore, any effect from the neutralizer has been normalized during calculation of the log_10_ reductions in titer. The cytotoxic effects of the mixtures of microbicides + chemical neutralizers on the host cells were evaluated as described in Supplementary Materials.

The inactivation results for BTC^®^ 835 and peracetic acid were analyzed by two-tailed T-test to determine the statistical significance of differences in the log_10_ reduction obtained for the three different viruses. To enable these comparisons, all log_10_ reduction values were considered complete. No statistical analyses were performed for sodium hypochlorite or ethanol, since any differences in log_10_ reduction obtained were attributed to differences in starting titer, as explained below in the Results section.

The virucidal efficacy of low pH treatment was determined as follows. For each replicate run, 1.0 mL of challenge virus (containing 5% horse serum for BVDV and 5% FBS for vaccinia virus and SARS-CoV-2 as organic load) was spiked into 19 mL of sterile deionized water. Under constant stirring, 0.5 N hydrochloric acid was added dropwise until the pH measured 3.0 ± 0.1. The resulting solution was held for either 10 min or 1 h. After the contact time, the solutions were neutralized 1:1 *v*/*v* with the appropriate chemical neutralizer (described above). The post-neutralized solution was 10-fold serially diluted in MEM + 2% horse serum for BVDV and MEM + 2% FBS for vaccinia virus and SARS-CoV-2 and inoculated onto appropriate host cells for each virus for determining the infectious titers using the TCID_50_ assay. The inoculated host cells were incubated at 36 °C with 5% CO_2_ for 5–11 days and scored for cytopathic effects. The infectious virus titers were calculated using the Spearman–Kärber formula [10].

The low pH inactivation results were analyzed by two-tailed T-test to determine the statistical significance of differences in the log_10_ reduction obtained after 10 min and after 60 min exposure for a given virus, and to compare the 10 min or 60 min exposure results between viruses. To enable these comparisons, all log_10_ reduction values were considered complete.

## 3. Results

In the side-by-side virucidal efficacy testing conducted for the three enveloped viruses (BVDV, vaccinia virus, and SARS-CoV-2) microbicide concentrations, pH, and contact times were selected to emphasize possible differences in susceptibility between the challenge viruses. A suspension-based virucidal efficacy test method (per ASTM E1052-20) was chosen to minimize variability from test substance application or carrier variation. The temperature was maintained at an ambient level (20–22 °C), such that differences in temperature (which can impact inactivation efficacy) could be ruled out in these studies. In each of the comparative inactivation efficacy assessments described below, three independent experimental runs were performed for each condition (a run consisted of a virus recovery control and a treated condition; three technical replicates were performed for each).

The exposure (contact) times evaluated for the four microbicides (sodium hypochlorite, ethanol, peracetic acid, and BTC^®^ 835) were kept quite short (1 min), while keeping the use concentrations similar to those typically used in the field. For the low pH inactivation study, the pH (3.0 to 3.1) and contact times evaluated are relevant to conditions used for viral clearance in biologic downstream processing [11].

### 3.1. Evaluation of Cytotoxic Effects of the Microbicides + Neutralizers

The results of the evaluation of the cytotoxic effects of the test microbicides and the neutralizers used to quench the virucidal activity of the test microbicides are displayed in Supplementary Materials Table S1. The cytotoxic effects of the mixture of microbicide + chemical neutralizer on the host cells, when found to be present, have been factored into the final log_10_ reduction results presented below.

### 3.2. Inactivation of Enveloped Viruses by Sodium Hypochlorite and Ethanol

**Figure 2:**
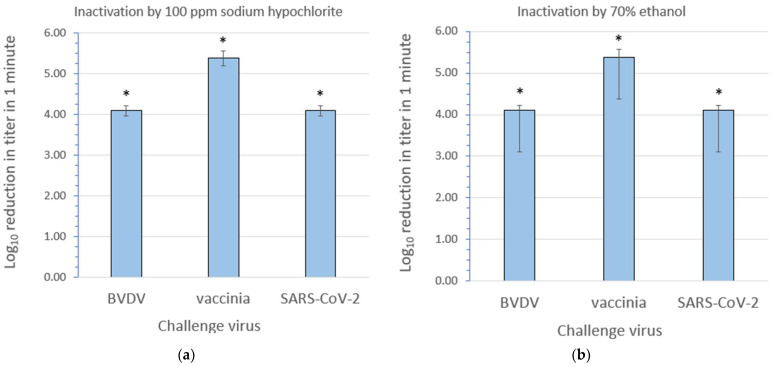
Inactivation of bovine viral diarrhea virus (BVDV), vaccinia virus, and severe acute respiratory syndrome coronavirus 2 (SARS-CoV-2) at room temperature by (**a**) 100 ppm sodium hypochlorite and (**b**) 70% ethanol. The values shown are the mean ± standard deviation for n = 3 independent replicate runs. * Inactivation was complete (no residual infectious virus was detected); therefore, the log_10_ reduction depicted is to be interpreted as “≥” the value shown.

The inactivation efficacy results for 100 ppm sodium hypochlorite and 70% ethanol are displayed in Figure 2a and Figure 2b, respectively. Additional details can be found in Supplementary Materials Tables S2–S5. Note that in each case, the inactivation of each virus by the microbicides was complete (no residual virus was detected post treatment). The differences in the log_10_ reduction in the titer between the three viruses post treatment do not reflect the differences in the susceptibility to the microbicide, but rather the differences in the starting titers (i.e., the titers for the virus recovery controls) for the three viruses (the mean starting titers for BVDV, vaccinia, and SARS-CoV-2 were 5.93, 6.61, and 5.93 log_10_ TCID_50_/mL, respectively). Therefore, statistical testing was not conducted. The mean ending titers following both sodium hypochlorite and ethanol treatment for BVDV, vaccinia, and SARS-CoV-2 were ≤1.83, ≤1.23, and ≤1.83 log_10_ TCID_50_/mL, respectively (in each case, no virus was detected following treatment).

### 3.3. Inactivation by BTC^®^ 835 and Peracetic Acid

**Figure 3:**
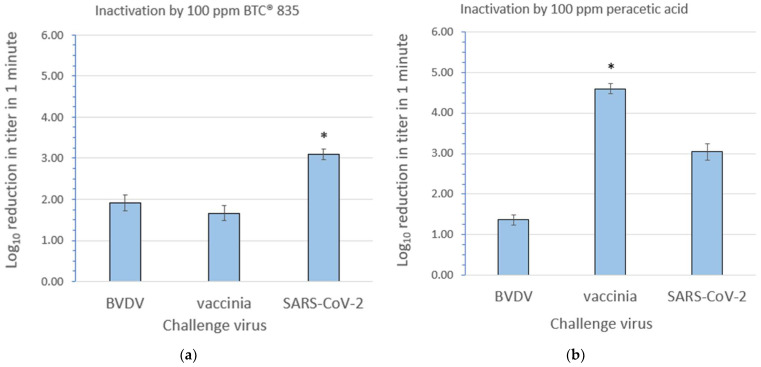
Inactivation of bovine viral diarrhea virus (BVDV), vaccinia virus, and severe acute respiratory syndrome coronavirus 2 (SARS-CoV-2) at room temperature by (**a**) 100 ppm BTC^®^ 835 and (**b**) 100 ppm peracetic acid. Values shown are the mean ± standard deviation for n = 3 independent replicate runs. * The inactivation of vaccinia virus by BTC^®^ 835 and of SARS-CoV-2 by peracetic acid was complete (no residual virus was detected); therefore, the log_10_ reductions depicted in these cases are to be interpreted as “≥” the values shown. In all other cases, inactivation was incomplete, meaning that residual infectious virus was detected. Two-tailed T-testing indicates that the differences between the log reduction values for peracetic acid for the three viruses are statistically significant (*p* < 0.05). For BTC^®^ 835, the log reduction values for BVDV and vaccinia virus are not statistically significant, but the differences between the log reduction values for BVDV and SARS-CoV-2 and for vaccinia virus and SARS-CoV-2 are statistically significant (*p* ≤ 0.05).

The inactivation efficacy results for 100 ppm BTC^®^ 835 and 100 ppm peracetic acid are displayed in Figure 3a,b, respectively. Additional details can be found in Supplementary Materials Tables S2–S5. The inactivation of SARS-CoV-2 by BTC^®^ 835 and of vaccinia virus by peracetic acid was complete (no residual virus was detected post treatment). For these specific cases, the log_10_ reductions in the titer for SARS-CoV-2 and vaccinia were, in part, determined by the starting titers (the mean starting titers for BVDV, vaccinia, and SARS-CoV-2 were 5.93, 6.61, and 5.93 log_10_ TCID_50_/mL, respectively). In all other cases, inactivation was incomplete, meaning that some residual infectious virus was detected. In these cases, differences in the log_10_ reduction solely reflect differences in the susceptibility of the viruses to the microbicides. The mean ending titers following BTC^®^ 835 treatment for BVDV, vaccinia, and SARS-CoV-2 were 4.01, 4.95, and 2.83 log_10_ TCID_50_/mL, respectively. The mean ending titers following peracetic acid treatment for BVDV, vaccinia, and SARS-CoV-2 were 4.55, ≤1.23 (no virus was detected post treatment), and 3.14 log_10_ TCID_50_/mL, respectively.

### 3.4. Inactivation by Low pH

**Figure 4:**
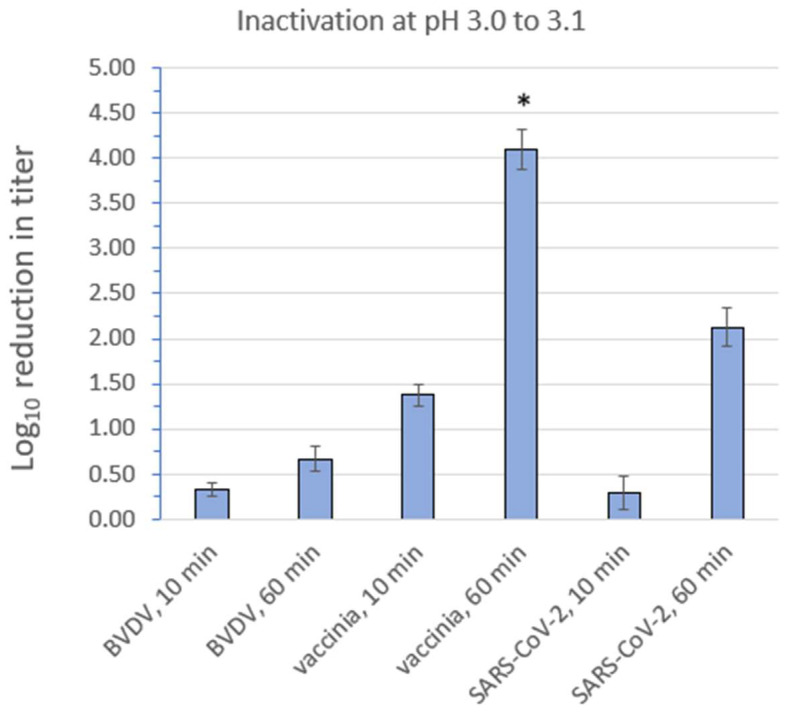
Inactivation of bovine viral diarrhea virus (BVDV), vaccinia virus, and severe acute respiratory syndrome coronavirus 2 (SARS-CoV-2) at room temperature by low pH (pH 3.0 to 3.1). Values shown are the mean ± standard deviation for n = 3 independent replicate runs. * The inactivation of vaccinia virus following 60 min exposure to low pH was complete (no residual infectious virus was detected); therefore, the log_10_ reduction depicted in this case is to be interpreted as “≥” the value shown. Two-tailed T-testing indicates that the differences between each 10 min reduction value and the corresponding 60 min reduction value are statistically significant (*p* ≤ 0.05). All pairwise comparisons between viruses were statistically significant (*p* ≤ 0.05), except for the 10 min exposure results for BVDV vs. SARS-CoV-2.

The efficacy of low pH inactivation is dependent upon the pH, temperature, and the exposure time. Since the pH was maintained at 3.0 to 3.1, and the temperature was maintained at an ambient level, the factors determining differences in log_10_ inactivation in this experiment were the exposure (contact) time and the virus. The results of the low pH inactivation study are displayed in Figure 4. Additional details can be found in Supplementary Materials Tables S2–S5.

For the 10 min low pH exposure study, the mean starting titers for BVDV, vaccinia, and SARS-CoV-2 were 5.55, 5.36, and 6.14 log_10_ TCID_50_/mL, respectively. The mean ending titers following 10 min of low pH treatment for BVDV, vaccinia, and SARS-CoV-2 were 5.22, 3.99, and 5.85 log_10_ TCID_50_/mL, respectively.

For the 60 min low pH exposure study, the mean starting titers for BVDV, vaccinia, and SARS-CoV-2 were 4.97, 5.32, and 5.26 log_10_ TCID_50_/mL, respectively. The mean ending titers following 60 min of low pH treatment for BVDV, vaccinia, and SARS-CoV-2 were 4.30, ≤1.23 (no virus was detected post treatment), and 3.13 log_10_ TCID_50_/mL, respectively.

It is apparent from Figure 4 that the flavivirus BVDV is much less susceptible to low pH inactivation than the poxvirus vaccinia virus, while the coronavirus SARS-CoV-2 displays a susceptibility intermediate between the other two enveloped viruses. In each case, the extent of inactivation was exposure time dependent, as expected.

## 4. Discussion

As mentioned previously, our aim in this work was to identify potential significant differences, if any, in the susceptibility of enveloped viruses to inactivation by low pH or by commonly used unformulated microbicidal active ingredients (sodium hypochlorite, ethanol, a quaternary ammonium compound, and peracetic acid). The three enveloped challenge viruses were the flavivirus BVDV, the poxvirus vaccina virus, and the coronavirus SARS-CoV-2. While the virucidal efficacy of some of these chemistries against enveloped viruses has been investigated previously in separate studies [5,6,12,13], a reliable comparison of the susceptibility of these viruses, in our view, must be conducted in side-by-side studies, where the same test method, test conditions (temperature, use concentrations), viral assay method, and analysts have been employed.

We selected SARS-CoV-2 as an example of a high-risk human pathogenic virus which first emerged in 2019 and which, for a period of time, was uncharacterized for microbicide susceptibility. In such cases, the United States Environmental Protection Agency (U.S. EPA) has instituted an interim policy based on the hierarchy of pathogen susceptibility to microbicides (mentioned previously) enabling non-label claims to be made regarding the efficacy of microbicides against such emerging viruses [14]. This Emerging Viral Pathogen (EVP) Policy has, in fact, been invoked in the past for pandemic influenza and for the Ebola virus, and most recently, proposed for SARS-CoV-2 [15]. During the COVID-19 pandemic, the appropriateness of the EVP policy was borne out, as SARS-CoV-2 proved to be readily inactivated by a variety of microbicidal active ingredients, as predicted by the hierarchy paradigm [6]. SARS-CoV-2 is a large (60–140 nm), enveloped, positive-sense single-stranded RNA virus of the *Coronaviridae* family [5].

Vaccinia virus is a large (270 × 350 nm), enveloped, double-stranded DNA virus of the *Poxviridae* family [16]. This virus was selected for use as a challenge virus in these studies since it has been stipulated as the representative enveloped virus to be used during virucidal efficacy testing in two European standardized methods, namely a suspension inactivation test (EN 14476) [17] and a surface inactivation test (EN 16777) [18]. Vaccinia virus is also stipulated as the European Tier 1 test virus for rapidly selecting disinfectants against emerging and re-emerging viral diseases [19]. One might assume that vaccinia virus was selected as the representative enveloped virus since it was considered a worst-case enveloped virus for susceptibility to microbicides. We find that the latter may not be true, as will be discussed below.

Bovine viral diarrhea virus is a virus of concern for bovine-derived raw materials, including fetal bovine serum [20]. It also has been used as a surrogate for the human pathogenic flavivirus hepatitis C virus (HCV) [21,22,23]. It is a medium-sized (40–60 nm), enveloped, single-stranded RNA virus of the *Flaviviridae* family [24]. The flavivirus was selected for inclusion in this study since we previously have concluded that BVDV represents a worst-case enveloped virus for low pH inactivation [25] due to its relatively high resistance. As BVDV is an enteric virus, this resistance to low pH is consistent with the requirement that it survive passage through the low pH environment of the stomach.

Our studies indicated that, indeed, BVDV was by far the most resistant of the three viruses to low pH treatment, with 60 min of exposure to pH 3.0 to 3.1 at room temperature causing a significantly lower log_10_ reduction than was observed for SARS-CoV-2 and vaccinia virus. SARS-CoV-2 has been evaluated previously for susceptibility to pH 3 treatment (60 min at room temperature) and found to be resistant [26,27]. Those results, taken together with ours and those of Darnell et al. using SARS-CoV [28] suggest that SARS-CoV-2 is only moderately susceptible to low pH. As SARS-CoV-2 causes both respiratory and enteric symptoms [29], this result may not be unexpected. Vaccinia virus was found to be the most susceptible of the three viruses to this treatment, both at 10 min exposure and at 60 min exposure. The mechanism of action of low pH for inactivating viruses has been suggested to involve the denaturing of the capsid protein, enabling proteases and nucleases to enter the capsid and subsequent damage to these macromolecules [25]. Interestingly, vaccinia virus, a double-stranded DNA virus, exhibited a notably higher susceptibility to low pH treatment than did SARS-CoV-2, which is a single-stranded RNA virus. This seems to suggest that, for enveloped viruses at least, the type of genomic material (DNA vs. RNA) may not directly affect viral susceptibility to low pH treatment. Similarly, Horst Ruppach of Charles River Laboratories [30] reported that the retrovirus HIV (human immunodeficiency virus) and BVDV (both being enveloped RNA viruses) were found to be much more resistant to low pH inactivation than MuLV (murine leukemia virus, a retrovirus with an RNA genome) and PRV (pseudorabies virus, a herpesvirus with a DNA genome). The implication of this is that the latter two viruses, which are typically included in viral clearance validation studies as model enveloped viruses, may not be the best choice. In Dr. Ruppach’s words, considering the low pH results as well as results from other viral purification steps, “These varying responses raise questions about using the same model viruses for all [viral clearance] steps, and a discussion should be initiated regarding how robust viral clearance can better be demonstrated.” [30]. We agree with this sentiment.

Going into these studies, we were not aware of comparative inactivation efficacy data for the four microbicides for these three challenge viruses. We should note at this time that the microbicides evaluated here were, in each case, non-formulated. The results that we have presented should, therefore, be extrapolated with caution to the same microbicidal actives in disinfectant formulations, as the latter often contain additives intended to increase efficacy and/or broaden the spectrum of pathogens that might be inactivated.

The hierarchy paradigm (Figure 1) predicts that sodium hypochlorite should inactivate not only enveloped viruses but also less susceptible pathogens, including non-enveloped viruses. We were, therefore, not surprised to observe that each of the three enveloped challenge viruses were completely inactivated by 100 ppm sodium hypochlorite in the relatively short contact time of 1 min (Figure 2a). Sodium hypochlorite is a stringent, broad-spectrum microbicidal active ingredient which is classified as an oxidizing agent. Its virucidal mechanism of action includes damage both to the viral nucleic acid and capsid [31,32]. The hierarchy paradigm also predicts that ethanol should inactivate enveloped viruses as well as pathogens with less susceptibility (Figure 1). Our results, again, indicated the complete inactivation of all three challenge viruses within 1 min (Figure 2b). The inactivation mechanism of ethanol, in the case of enveloped viruses, primarily involves the disruption of the lipid envelope [32,33].

The unformulated quaternary ammonium compound BTC^®^ 835 was predicted, on the basis of the hierarchy paradigm (Figure 1), to inactivate only the most susceptible pathogen types, including enveloped viruses. The mechanism of action of QAC, in general, primarily involves the disruption of the viral lipid envelope [32,34]. Our results indicate that even among enveloped viruses, significant differences in the inactivation efficacy may be observed with this microbicide. For instance, SARS-CoV-2 was found (Figure 3a) to be significantly more susceptible to BTC^®^ 835 than BVDV or vaccinia virus, while BVDV and vaccinia virus displayed a similar susceptibility. Peracetic acid is classified as a peroxygen, and as such, was expected to represent a broad-spectrum microbicide. Investigators have reported that the inactivation of viruses by peracetic acid is mediated primarily through targeting susceptible amino acids on capsid proteins and not through damage to viral genomes [12,32]. We found it somewhat surprising, therefore, that our results indicated significant differences in inactivation efficacy for the three challenge enveloped viruses. The order of susceptibility for this microbicide was found to be vaccinia virus > SARS-CoV-2 > BVDV.

At this time, we are unable to postulate the reasons for the differences in the susceptibility of the three challenge viruses to certain microbicides evaluated (e.g., peracetic acid and BTC^®^ 835). It is likely, of course, that higher concentrations of these two microbicidal actives might result in the complete inactivation of all three viruses within the short contact time period evaluated, or alternatively by the same concentration (100 ppm) administered for longer contact times. It is of interest, though, that for whatever reason, vaccinia virus was not found to be the worst-case enveloped virus (i.e., least susceptible) for any of the microbicides evaluated or for low pH treatment. BVDV, on the other hand, clearly represented the worst-case enveloped virus for low pH treatment and for 100 ppm peracetic acid. The use of BVDV as a challenge virus (in addition to vaccinia virus) is currently indicated in the Verbund für Angewandte Hygiene e.V (Association for Applied Hygiene) Annex V (Requirements for virucidal activity) for testing products with oxidative activity [35]. Our results with peracetic acid (an oxidizing agent) are consistent with, and confirm the reasoning behind, the addition of BVDV as a challenge virus for such products. Hepatitis B virus (HBV) has been considered, according to some literature, to be among the most difficult of the human blood-borne enveloped viruses to inactivate [36]. This virus has not been included in the current study due to the lack of suitable cell-based infectivity assays for this specific virus (not the duck hepatitis B virus, which has been used as a surrogate for HBV) with which to conduct viral inactivation studies.

## 5. Conclusions

The results of our study indicate that significant differences in the susceptibility of enveloped viruses to commonly used chemical inactivation approaches (unformulated microbicidal actives and low pH treatment) exist, under defined conditions. While the underlying reasons for the differences are not clear, the implication of the finding is that a single enveloped virus, such as vaccinia virus, may not represent a worst-case challenge virus for all inactivation approaches. If it is necessary to select a single enveloped virus for validating virucidal efficacy or purification process viral clearance, then perhaps BVDV, a virus typically available in viral safety testing laboratories, should be considered.

## Data Availability

Data are contained within the article and Supplementary Materials.

## Supplementary Materials

The following supporting information can be downloaded at https://www.mdpi.com/article/10.3390/microorganisms12030535/s1, Table S1: Interference and cytotoxicity testing; Table S2: Titer results—Bovine Viral Diarrhea Virus (BVDV); Table S3: Titer results—Vaccinia virus; Table S4: Titer results—Severe Acute Respiratory Syndrome Coronavirus 2 (SARS-CoV-2) (COVID-19 virus); Table S5: Viral log reduction summary.
