# Supplementary material for: Differing Susceptibilities to Certain Microbicidal Chemistries among Three Representative Enveloped Viruses
Source: Microorganisms. 2024 Mar 7;12(3):535. doi: 10.3390/microorganisms12030535 (PMC10975453; doi:10.3390/microorganisms12030535)
Supplement: Supplementary file 1 [file microorganisms-12-00535-s001.zip › microorganisms-2800420-supplementary.pdf]

Supplementary Materials

Differing susceptibilities to microbicidal chemistries among three representative enveloped viruses

Tanya Kapes, Charles Quinn, Andrew Eli Cragun, Taylor House, Raymond W. Nims, and S. Steve Zhou

1. Evaluation of the cytotoxic effects of the test microbicides and the neutralizers used.

For the neutralizer effectiveness/viral interference and cytotoxicity controls, one replicate was performed for each test substance and each virus. This control was performed in the same manner as in the efficacy test, using dilution media in place of virus. Samples were neutralized 1:1 with their respective neutralizer and this post-neutralized sample (considered undilute) was divided into two portions. Both portions were ten-fold serially diluted in dilution medium. For the cytotoxicity control, these dilutions were inoculated onto host cells. For the neutralization effectiveness/viral interference testing (NE/VI), the diluted samples (4.5 mL) were spiked with 100 µL of low titered virus (containing no more than approximately 5,000 $TCID_{50}$ units) and were held for at least the contact time (but no longer 30 minutes). After holding, the dilutions were plated onto host cells.

Table S1. Interference and cytotoxicity testing

Neutralizer Effectiveness/Viral Interference (NE/VI) and Cytotoxicity (CT) Controls

100 ppm Quaternary Ammonium Compound (BTC® 835) (Vaccinia Virus)

Dilution*	NE/VI	CT
10^{-1}	Cytotoxicity Observed	Cytotoxicity Observed
10^{-2}	Cytotoxicity Observed	Cytotoxicity Observed
10^{-3}	virus detected in all inoculated wells	no virus detected in all inoculated wells

* Dilution refers to the fold of the dilution from the neutralized sample.

100 ppm Quaternary Ammonium Compound (BTC® 835) (BVDV/SARS-CoV-2)

Dilution*	NE/VI	CT
10^{-1}	Cytotoxicity Observed	Cytotoxicity Observed
10^{-2}	virus detected in all inoculated wells	no virus detected in all inoculated wells
10^{-3}	virus detected in all inoculated wells	no virus detected in all inoculated wells

* Dilution refers to the fold of the dilution from the neutralized sample.

100 ppm Sodium Hypochlorite, 70% Ethanol, 100 ppm Peracetic Acid, and Low pH by Hydrochloric Acid (pH 3.0 - 3.1) (all viruses)

Dilution*	NE/VI	CT
10^{-1}	virus detected in all inoculated wells	no virus detected in all inoculated wells
10^{-2}	virus detected in all inoculated wells	no virus detected in all inoculated wells
10^{-3}	virus detected in all inoculated wells	no virus detected in all inoculated wells

* Dilution refers to the fold of the dilution from the neutralized sample.

2. Efficacy testing tables

Table S2. Titer Results - Bovine Viral Diarrhea Virus (BVDV)

Sample	Contact Time	Replicate	Titer ($Log_{10}TCID_{50}/mL$)	Volume (mL)	Volume Correction [a]	Viral Load ($Log_{10}TCID_{50}$)
Virus Stock Titer Control (2/22/23)	N/A	N/A	6.55 ± 0.16	-	-	-
Virus Stock Titer Control (3/16/23)			6.80 ± 0.17	-	-	-
Virus Stock Titer Control (9/14/23)			7.05 ± 0.16	-	-	-
Virus Stock Titer Control (10/05/23)			6.05 ± 0.16	-	-	-
Cell Viability Control			no virus was detected, cells remained viable; media was sterile			
Testing performed 02/22/23						
Virus Recovery Control	60 seconds	Rep. 1	5.93 ± 0.12	3	2	6.71 ± 0.12
		Rep. 2	6.05 ± 0.16	3	2	6.83 ± 0.16
		Rep. 3	5.80 ± 0.00	3	2	6.58 ± 0.00
		Average ± 95% CI				6.70 ± 0.20
100 ppm Sodium Hypochlorite		Rep. 1	≤ 1.83 *	3	2	≤ 2.61
		Rep. 2	≤ 1.83 *	3	2	≤ 2.61
		Rep. 3	≤ 1.83 *	3	2	≤ 2.61
		Average ± 95% CI				≤ 2.61 ± 0.00
70% Ethanol		Rep. 1	≤ 1.83 *	3	2	≤ 2.61
		Rep. 2	≤ 1.83 *	3	2	≤ 2.61
		Rep. 3	≤ 1.83 *	3	2	≤ 2.61
		Average ± 95% CI				≤ 2.61 ± 0.00
100 ppm Quaternary Ammonium Compound (BTC® 835)		Rep. 1	4.05 ± 0.16	3	2	4.83 ± 0.16
		Rep. 2	3.93 ± 0.12	3	2	4.71 ± 0.12
		Rep. 3	4.05 ± 0.16	3	2	4.83 ± 0.16
		Average ± 95% CI				4.79 ± 0.26
Testing performed 03/16/23						
Virus Recovery Control	60 seconds	Rep. 1	5.93 ± 0.12	3	2	6.71 ± 0.12
		Rep. 2	6.05 ± 0.22	3	2	6.83 ± 0.22
		Rep. 3	5.80 ± 0.00	3	2	6.58 ± 0.00
		Average ± 95% CI				6.70 ± 0.25
100 ppm Peracetic acid		Rep. 1	4.43 ± 0.22	3	2	5.21 ± 0.22
		Rep. 2	4.68 ± 0.20	3	2	5.46 ± 0.20
		Rep. 3	4.55 ± 0.16	3	2	5.33 ± 0.16
		Average ± 95% CI				5.33 ± 0.34
Testing performed 09/14/23						
Virus Recovery Control	10 minutes	Rep. 1	5.43 ± 0.18	20	2	7.03 ± 0.18
		Rep. 2	5.55 ± 0.22	20	2	7.15 ± 0.22
		Rep. 3	5.68 ± 0.24	20	2	7.28 ± 0.24
		Average ± 95% CI				7.16 ± 0.37
Low pH by Hydrochloric Acid (pH 3.0 - 3.1)		Rep. 1	5.18 ± 0.18	20	2	6.78 ± 0.18
		Rep. 2	5.18 ± 0.18	20	2	6.78 ± 0.18
		Rep. 3	5.30 ± 0.19	20	2	6.90 ± 0.19
		Average ± 95% CI				6.82 ± 0.32
Testing performed 10/05/23						
Virus Recovery Control	1 hour	Rep. 1	5.05 ± 0.16	20	2	6.65 ± 0.16
		Rep. 2	5.05 ± 0.16	20	2	6.65 ± 0.16
		Rep. 3	4.80 ± 0.17	20	2	6.40 ± 0.17
		Average ± 95% CI				6.57 ± 0.28
Low pH by Hydrochloric Acid (pH 3.0 - 3.1)		Rep. 1	4.30 ± 0.19	20	2	5.90 ± 0.19
		Rep. 2	4.30 ± 0.19	20	2	5.90 ± 0.19
		Rep. 3	4.30 ± 0.22	20	2	5.90 ± 0.22
		Average ± 95% CI				5.90 ± 0.35

[a] Volume correction accounts for the neutralization of the sample post contact time.
* No virus detected; the theoretical titer was determined based on the Poisson Distribution.

Table S3. Titer Results - Vaccinia virus

Sample	Contact Time	Replicate	Titer ($Log_{10}TCID_{50}$/mL)	Volume (mL)	Volume Correction [a]	Viral Load ($Log_{10}TCID_{50}$)
Virus Stock Titer Control (2/22/23)	N/A	N/A	7.57 ± 0.22	-	-	-
Virus Stock Titer Control (3/16/23)			6.82 ± 0.18	-	-	-
Virus Stock Titer Control (9/14/23)			7.07 ± 0.20	-	-	-
Virus Stock Titer Control (10/05/23)			6.70 ± 0.19	-	-	-
Cell Viability Control			no virus was detected, cells remained viable; media was sterile			
Testing performed 02/22/23						
Virus Recovery Control	60 seconds	Rep. 1	6.57 ± 0.18	3	2	7.35 ± 0.18
		Rep. 2	6.82 ± 0.18	3	2	7.60 ± 0.18
		Rep. 3	6.45 ± 0.22	3	2	7.23 ± 0.22
		Average ± 95% CI				7.39 ± 0.34
100 ppm Sodium Hypochlorite		Rep. 1	≤ 1.23 *	3	2	≤ 2.01
		Rep. 2	≤ 1.23 *	3	2	≤ 2.01
		Rep. 3	≤ 1.23 *	3	2	≤ 2.01
		Average ± 95% CI				≤ 2.01 ± 0.00
70% Ethanol		Rep. 1	≤ 1.23 *	3	2	≤ 2.01
		Rep. 2	≤ 1.23 *	3	2	≤ 2.01
		Rep. 3	≤ 1.23 *	3	2	≤ 2.01
		Average ± 95% CI				≤ 2.01 ± 0.00
100 ppm Quaternary Ammonium Compound (BTC® 835)		Rep. 1	5.07 ± 0.24	3	2	5.85 ± 0.24
		Rep. 2	4.95 ± 0.22	3	2	5.73 ± 0.22
		Rep. 3	4.82 ± 0.18	3	2	5.60 ± 0.18
		Average ± 95% CI				5.72 ± 0.37
Testing performed 03/16/23						
Virus Recovery Control	60 seconds	Rep. 1	5.70 ± 0.19	3	2	6.48 ± 0.19
		Rep. 2	5.95 ± 0.16	3	2	6.73 ± 0.16
		Rep. 3	5.82 ± 0.18	3	2	6.60 ± 0.18
		Average ± 95% CI				6.60 ± 0.31
100 ppm Peracetic acid		Rep. 1	≤ 1.23 *	3	2	≤ 2.01
		Rep. 2	≤ 1.23 *	3	2	≤ 2.01
		Rep. 3	≤ 1.23 *	3	2	≤ 2.01
		Average ± 95% CI				≤ 2.01 ± 0.00
Testing performed 09/14/23						
Virus Recovery Control	10 minutes	Rep. 1	5.57 ± 0.16	20	2	7.17 ± 0.16
		Rep. 2	5.45 ± 0.22	20	2	7.05 ± 0.22
		Rep. 3	5.07 ± 0.28	20	2	6.67 ± 0.28
		Average ± 95% CI				6.97 ± 0.39
Low pH by Hydrochloric Acid (pH 3.0 - 3.1)		Rep. 1	4.07 ± 0.12	20	2	5.67 ± 0.12
		Rep. 2	4.20 ± 0.00	20	2	5.80 ± 0.00
		Rep. 3	3.70 ± 0.19	20	2	5.30 ± 0.19
		Average ± 95% CI				5.59 ± 0.22
Testing performed 10/05/23						
Virus Recovery Control	1 hour	Rep. 1	5.45 ± 0.16	20	2	7.05 ± 0.16
		Rep. 2	5.45 ± 0.22	20	2	7.05 ± 0.22
		Rep. 3	5.07 ± 0.28	20	2	6.67 ± 0.28
		Average ± 95% CI				6.93 ± 0.39
Low pH by Hydrochloric Acid (pH 3.0 - 3.1)		Rep. 1	≤ 1.23 *	20	2	≤ 2.83
		Rep. 2	≤ 1.23 *	20	2	≤ 2.83
		Rep. 3	≤ 1.23 *	20	2	≤ 2.83
		Average ± 95% CI				≤ 2.83 ± 0.00

[a] Volume correction accounts for the neutralization of the sample post contact time.

* No virus detected; the theoretical titer was determined based on the Poisson Distribution.

Table S4. Titer Results - Severe Acute Respiratory Syndrome Coronavirus 2 (SARS-CoV-2) (COVID-19 virus)

Sample	Contact Time	Replicate	Titer ($Log_{10}TCID_{50}$/mL)	Volume (mL)	Volume Correction [a]	Viral Load ($Log_{10}TCID_{50}$)
Virus Stock Titer Control (2/22/23)	N/A	N/A	7.05 ± 0.16	-	-	-
Virus Stock Titer Control (3/16/23)			6.68 ± 0.12	-	-	-
Virus Stock Titer Control (09/14/23)			7.80 ± 0.17	-	-	-
Virus Stock Titer Control (10/05/23)			6.18 ± 0.18	-	-	-
Cell Viability Control			no virus was detected, cells remained viable; media was sterile			
Testing performed 02/22/23						
Virus Recovery Control	60 seconds	Rep. 1	6.05 ± 0.16	3	2	6.83 ± 0.16
		Rep. 2	5.80 ± 0.00	3	2	6.58 ± 0.00
		Rep. 3	5.93 ± 0.12	3	2	6.71 ± 0.12
		Average ± 95% CI				6.70 ± 0.20
100 ppm Sodium Hypochlorite		Rep. 1	≤ 1.83 *	3	2	≤ 2.61
		Rep. 2	≤ 1.83 *	3	2	≤ 2.61
		Rep. 3	≤ 1.83 *	3	2	≤ 2.61
		Average ± 95% CI				≤ 2.61 ± 0.00
70% Ethanol		Rep. 1	≤ 1.83 *	3	2	≤ 2.61
		Rep. 2	≤ 1.83 *	3	2	≤ 2.61
		Rep. 3	≤ 1.83 *	3	2	≤ 2.61
		Average ± 95% CI				≤ 2.61 ± 0.00
100 ppm Quaternary Ammonium Compound (BTC® 835)		Rep. 1	2.83 *	3	2	≤ 3.61
		Rep. 2	2.83 *	3	2	≤ 3.61
		Rep. 3	2.83 *	3	2	≤ 3.61
		Average ± 95% CI				≤ 3.61 ± 0.00
Testing performed 03/16/23						
Virus Recovery Control	60 seconds	Rep. 1	6.30 ± 0.19	3	2	7.08 ± 0.19
		Rep. 2	6.18 ± 0.18	3	2	6.96 ± 0.18
		Rep. 3	6.05 ± 0.16	3	2	6.83 ± 0.16
		Average ± 95% CI				6.95 ± 0.31
100 ppm Peracetic acid		Rep. 1	3.05 ± 0.16	3	2	3.83 ± 0.16
		Rep. 2	3.18 ± 0.18	3	2	3.96 ± 0.18
		Rep. 3	3.18 ± 0.18	3	2	3.96 ± 0.18
		Average ± 95% CI				3.91 ± 0.30
Testing performed 09/14/23						
Virus Recovery Control	10 minutes	Rep. 1	6.43 ± 0.18	20	2	8.03 ± 0.18
		Rep. 2	6.05 ± 0.24	20	2	7.65 ± 0.24
		Rep. 3	5.93 ± 0.20	20	2	7.53 ± 0.20
		Average ± 95% CI				7.74 ± 0.36
Low pH by Hydrochloric Acid (pH 3.0 - 3.1)		Rep. 1	5.93 ± 0.20	20	2	7.53 ± 0.20
		Rep. 2	5.93 ± 0.12	20	2	7.53 ± 0.12
		Rep. 3	5.68 ± 0.24	20	2	7.28 ± 0.24
		Average ± 95% CI				7.45 ± 0.33
Testing performed 10/05/23						
Virus Recovery Control	1 hour	Rep. 1	5.55 ± 0.22	20	2	7.15 ± 0.22
		Rep. 2	5.30 ± 0.19	20	2	6.90 ± 0.19
		Rep. 3	4.93 ± 0.12	20	2	6.53 ± 0.12
		Average ± 95% CI				6.86 ± 0.31
Low pH by Hydrochloric Acid (pH 3.0 - 3.1)		Rep. 1	3.30 ± 0.19	20	2	4.90 ± 0.19
		Rep. 2	3.05 ± 0.16	20	2	4.65 ± 0.16
		Rep. 3	3.05 ± 0.16	20	2	4.65 ± 0.16
		Average ± 95% CI				4.74 ± 0.30

[a] Volume correction accounts for the neutralization of the sample post contact time.

* No virus detected; the theoretical titer was determined based on the Poisson Distribution.

Table S5. Viral Reduction

Chemical Treatment	Contact Time	Log$_{10}$ Reduction ± 95% CI*		
		BVDV	Vaccinia	SARS-CoV-2
100 ppm Sodium Hypochlorite	60 seconds	≥ 4.10 ± 0.20	≥ 5.38 ± 0.34	≥ 4.10 ± 0.20
70% Ethanol	60 seconds	≥ 4.10 ± 0.20	≥ 5.38 ± 0.34	≥ 4.10 ± 0.20
100 ppm Peracetic acid	60 seconds	1.37 ± 0.42	≥ 4.59 ± 0.31	3.04 ± 0.43
100 ppm Quaternary Ammonium Compound (BTC® 835)	60 seconds	1.92 ± 0.32	1.67 ± 0.50	≥ 3.10 ± 0.20
Low pH by Hydrochloric Acid (pH 3.0 - 3.1)	10 minutes	0.33 ± 0.49	1.37 ± 0.45	0.29 ± 0.49
Low pH by Hydrochloric Acid (pH 3.0 - 3.1)	1 hour	0.67 ± 0.45	≥ 4.09 ± 0.39	2.13 ± 0.43

* Average from three replicate experiments

"≥" denotes a complete inactivation of virus

Summary of Experiment

In testing for 100 ppm Sodium Hypochlorite, 70% Ethanol, 100 ppm Peracetic acid, and 100 ppm Quaternary Ammonium Compound (BTC® 835), 0.3 mL of challenge virus containing 5% serum was spiked into 2.7 mL of the test substance and held for 1 minute. After the contact time, the reaction mixture was neutralized 1:1 with an appropriate chemical neutralizer.

In testing for Low pH by hydrochloric acid, 1.0 mL of challenge virus containing 5% serum was spiked into 19 mL of sterile deionized water and the initial pH was measured. Under constant stirring, 0.5N Hydrochloric acid was added dropwise until the pH measured 3.0 ± 0.1. This mixture was held for either 10 minutes or 1 hour. After the contact time, the reaction mixture was neutralized 1:1 with an appropriate chemical neutralizer.
